# The Effect of Environmental Factors, Health Behaviors, and Psychosocial Aspects on Allergic Diseases in Korean Adolescents

**DOI:** 10.3390/medicina61040727

**Published:** 2025-04-14

**Authors:** Hwa-jin Lee, You Hoon Jeon

**Affiliations:** 1 Department of Nursing Science, College of Medicine, Kyungbuk College, Yeongju 36133, Republic of Korea; owelri9@naver.com; 2 Department of Pediatrics, Hallym University Dongtan Sacred Heart Hospital, Hwaseong 18450, Republic of Korea

**Keywords:** adolescent, atopic dermatitis, allergic rhinitis, asthma, social environment, psychological stress

## Abstract

*Background and Objectives*: Adolescence is a critical period of physical and mental development, yet allergic diseases are often poorly managed. Factors such as sleep deprivation, obesity, smoking, and mental stress can worsen allergic conditions and complicate treatment. This study examines the environmental, behavioral, and psychosocial factors influencing allergic diseases in Korean adolescents using data from the Korean National Health and Nutrition Examination Survey (KNHNES). *Materials and Methods*: From the 25,534 participants in the Fifth KNHNES, 1630 adolescents (aged 13–18 years) were selected. We analyzed demographic and lifestyle factors, including gender, age, housing type, family size, economic status, obesity, tobacco and alcohol use, sleep duration, and physical activity. Psychosocial factors such as stress perception, suicidal ideation, depressive symptoms, mental health counseling, and self-rated health were also examined. *Results*: The prevalence rates of allergic diseases were 23% for allergic rhinitis (AR), 11% for atopic dermatitis (AD), and 9.8% for asthma (AS), with 35.8% of adolescents having at least one allergic condition. Smoking was significantly associated with AS (odds ratio [OR] 1.753, *p* = 0.006), while shorter sleep durations increased AR risk (*p* = 0.000). Male adolescents had a lower risk of AD (OR 0.706, *p* = 0.046), and high economic status was inversely correlated with AD (OR 0.445, *p* = 0.006). Positive self-rated health was linked to lower AS risk (OR 0.447, *p* = 0.000). AR was significantly associated with male gender (OR 1.391, *p* = 0.045), high economic status (OR 1.784, *p* = 0.026), and high stress perception (OR 1.479, *p* = 0.013). *Conclusions*: Low self-rated health and high stress perception have been identified as risk factors for allergic diseases during adolescence. Integrating psychosocial counseling with medical treatment may improve management and outcomes.

## 1. Introduction

Allergic diseases are chronic conditions that greatly affect an individual’s quality of life. It has been reported that atopic dermatitis (AD) also affects an individual’s quality of life more than chronic diseases such as diabetes, kidney disease, and epilepsy because of the associated negative effects of lack of sleep, bullying, difficulty in participating in sports, lack of confidence, and depression [1]. Some studies have shown that allergic diseases, such as allergic rhinitis (AR) and asthma (AS), affect sleep and school grades [2,3,4].

Adolescence is a period of rapid physical development and a period of high stress and emotional changes, which can lead to mental health issues. Adolescence is an important period in which one’s lifestyle, such as eating habits, nutrition, and exercise, are established, and awareness of health arises [5,6]. However, it is also a time when allergic disease, which is one of the most common chronic diseases among adolescents, can be poorly managed. If accompanying an allergic disorder, factors such as sleep duration, obesity, smoking, and mental stress can worsen the disease and make it more difficult to treat. There has been little research into the relationship between allergic diseases in adolescents and environmental factors, health behaviors, and psychosocial factors.

Therefore, the current study aims to identify the factors influencing allergic diseases in Korean adolescents by using data from the Korean National Health and Nutrition Survey (KNHNES) and to provide strategies for promoting the health of adolescents with allergic diseases.

## 2. Materials and Methods

### 2.1. Study Design

Raw data from the fifth (2010–2012) KNHNES were used. Out of a total of three years of raw data, data from teenagers aged 13 to 18 were extracted and analyzed. Environmental factors, health behaviors, and psychological and social factors were analyzed based on the presence and absence of AD, AR, and AS.

(1) Environmental factors

The environmental factors included residence, housing type, number of households, and economic level. The ages were divided into 13–15 (middle school age) and 16–18 (high school age). The question about the number of household members was as follows: “How many members of the family are currently living together?” The subjects were asked whether their housing type was a general house or an apartment. Residential areas were classified into urban and rural areas. The economic level classified income as “lower, lower-middle, higher-middle, or higher”.

(2) Health behaviors

Health behaviors included obesity, vaccination, smoking, drinking, sleeping hours, and physical activity. Alcohol drinking (and likewise, tobacco smoking) was defined as drinking (smoking) at least once within a month, and non-drinking (non-smoking) was defined as not drinking (smoking) in the last year. The vaccination question asked whether they had been vaccinated against influenza (seasonal flu) in the past year, which was measured with a “yes/no” response. Sleep time was divided into more than seven hours/less than seven hours using the following question: “How many hours do you usually sleep a day?”

Intense exercise was defined as cases in which vigorous physical activities, which are more difficult than usual, had been practiced for more than 20 min a day and more than three times a week for the past week. Moderate-intensity exercise was defined as cases that are slightly more difficult than usual for more than 30 min a day and more than five times a week. Walking exercise was counted when performed for more than 30 min a day and more than five times a week. Muscle strength exercise was defined as doing push-ups, sit-ups, dumbbells, weights, and iron bars for more than two days a week, and flexibility exercise was defined as more than two days of flexibility exercises, such as stretching and bare-handed gymnastics. Body mass index (BMI) was calculated from the weight and height measured directly during the survey, and a BMI above 25 kg/m^2^ was classified as obese.

(3) Psychosocial factors

Psychological and social factors such as stress perception, suicidal thoughts, depression experience, activity restriction, experience of counseling for mental health issues, and self-rated health were analyzed. Stress awareness was divided into several levels of stress in everyday life, reclassifying “very much”, “quite a lot”, and “a little bit” as “stress recognition” and “no or little stress” as “stress non-recognition.”

The question regarding suicide asked if the subject had ever thought of committing suicide in the last year. As for depression, the question asked whether or not the participant had felt sad or hopeless enough to have these feelings interfere with one’s daily life for more than two consecutive weeks over the past year. The question of mental counseling asked whether they had visited a clinic or received counseling over the phone or the internet because of mental problems in the past year. When asked about their usual health conditions, self-rated health reclassified “very good” and “good” to “good responses” and “normal”, “bad” and “very bad” to “bad responses”. Activity restriction was assessed using the following question: “Have you been absent from school because of illness in the last month?”

### 2.2. Statistical Analysis

The statistical analysis used SAS software (version 9.3; SAS Institute, Cary, NC, USA). The analysis was conducted by applying the sample weights provided by the KNHNES so that we could predict the entire nation’s adolescents using this population group. The statistical significance test was based on a significance level of <0.05, and the specific statistical analysis was performed as follows.

(1) The prevalence of allergic diseases in adolescents (AD, AR, AS) was analyzed using the frequency, percentage, and standard error.
(2) The difference between environmental factors, health behaviors, and psychosocial factors according to the presence and absence of allergic diseases in adolescents was compared with a chi-square (χ^2^) test.
(3) To identify the explanatory power of the variable showing the difference between the prevalence of allergic diseases in adolescents, a binary logistic regression was conducted by adjusting the age and sex and using allergic disease as the dependent variable.
(4) To identify the risk factors for allergic diseases in adolescents, a multiple logistic regression was performed, using variables that showed significant differences in the χ^2^ test as predictors.

### 2.3. Ethics Statement

The KNHNES was conducted with the approval of the Research Ethics Review Committee of the Korea Centers for Disease Control and Prevention (Approval Number: 2010-02CON-21-C 2011-02CON-06-C 2012-01EXP-01-2C). 

## 3. Results

### 3.1. Characteristics of Allergic Diseases in Adolescents

A total of 1630 adolescents who completed the survey were enrolled in the current study. Among them, 35.8% had one or more allergic disease. Among the studied allergic diseases, AR had the highest prevalence rate of 23.0%, while AD and AS had prevalence rates of 11.2% and 9.8%, respectively. The age-based prevalence of allergic diseases ranked in the order of AR, AD, then AS for both age group ranges of 13–15 and 16–18, with no difference between the two groups being found.

### 3.2. Relationship Between Environmental Factors and Allergic Diseases in Korean Adolescents

In the case of male students, more teenagers were diagnosed with AR (*p* = 0.011). There were more adolescents diagnosed with AS when the economic level was “lower” (*p* = 0.004), while there were more adolescents diagnosed with AR when the economic level was “higher” (*p* = 0.011). Adolescents diagnosed with AS were found to be more likely to live in general housing than in apartments, a difference found to be statistically significant (72.7% vs. 27.3%, *p* = 0.048). Students with AD had a significant difference in their number of family members compared with students without AD (*p* = 0.022). In addition, 19.4% of adolescents with AD had more than five family members, while 30.3% of adolescents without AD had more than five family members. The data are shown in Table 1.

### 3.3. Relationship Between Health Behaviors and Allergic Diseases in Korean Adolescents

The alcohol drinking rate was 39.7% in adolescents with AS, 34.9% in AD, and 29.5% in AR, indicating that adolescents diagnosed with AS had the highest drinking rate. However, alcohol drinking and allergic diseases were found to have no statistically significant relationship. The tobacco smoking rate was 20.6% for AS, 14.4% for AD, and 13.9% for AR in adolescents. Adolescents with AS were significantly more likely to smoke than those without AS (20.6% vs. 12.5%, *p* = 0.034).

For intense exercise more than three times a week for more than 20 min, there was a prevalence rate of 33.3% in adolescents with AS, 31.4% for AR, and 23.9% for AD. There was no statistically significant relationship between exercise volume or form and allergic diseases. For amount of sleep, 61.1% of the adolescents with AS, 64.0% with AD, and 72% with AR reported sleeping less than seven hours a day on average. Adolescents diagnosed with AR were significantly more likely to sleep for less than seven hours a day than adolescents who did not have AR (72% vs. 62.1%, *p* = 0.002).

There were more obese adolescents with AS than those without AS, but this was not statistically significant (*p* = 0.056). Adolescents with AR were likely to be obese than those without AR, but this was not significant (*p* = 0.057). Among the adolescents with AD, 88.5% had a low 25(OH) vitamin D concentration (less than 20 ng/mL); this level was 82.2% in adolescents with AS and 76.5% in those with AR. However, the vitamin D levels in adolescents showed no significant difference (Table 2).

### 3.4. Relationship Between Psychosocial Factors and Allergic Diseases in Korean Adolescents

As for whether there were any absences from school within the past month, the absence rate was 6.2% for AS, 5.0% for AR, and 3.3% for AD. However, there were no significant differences in the presence or absence of each allergic disease. Those who said they felt stressed included 29.8% of adolescents with AD and 28.6% with AS. Among the adolescents with AR, 30.8% felt stressed, which was significantly higher than in adolescents without AR (25.1%) (*p* = 0.047). Here, 18.6% of adolescents with AS, 17.0% with AR, and 14.8% with AD had thought of committing suicide. For those stating they experienced depression, 12.8% had AR, 10.8% had AS, and 7.5% had AD. Psychiatric counseling occurred in 5.8% of those with AD, 5.0% of those with AR, and 4.9% of those with AS, but these results were not statistically significant. Negative responses to self-rated health status were 58.9% for teenagers with AS, followed by 45.7% for AR and 43.7% for AD. Negative perceptions of subjective health conditions were statistically significantly higher in adolescents with AS than those without AS (58.9% vs. 38.7%, *p* = 0.000) (Table 3).

### 3.5. Odds Ratio of the Risk Factors Using a Binary Logistic Regression in Korean Adolescents’ Allergic Diseases

An age- and gender-adjusted binary logistic regression was conducted to identify the effects of environmental factors, health behaviors, and psychosocial factors on allergic diseases in adolescents. For the male gender, the odds ratio (OR) was statistically significant at 1.441 (95% CI [1.082–1.916] for AR (*p* = 0.036). For economic status, the OR for AR prevalence was 1.965 times the “higher” economic level (95%CI [1.129–3.419]) compared with the “lower” economic level. AS had a high prevalence rate when the economic level was low (*p* = 0.015). AS prevalence was lower in those living in apartments compared with those living in general houses (OR 0.679, 95% CI [0.46–1.00], *p* = 0.048). When the number of family members was small, the OR of AD was high, indicating a statistically significant difference (*p* = 0.006) (Table 4). For adolescents who smoke, the diagnosis of AS was higher, at an OR of 1.753 (95% CI [0.974–3.155]) (*p* = 0.006). The OR of AR was higher in the case of sleeping less than seven hours, and there was a statistically significant difference (*p* = 0.000) (Table 5). In the case of “I feel stress”, the OR for AR was 1.399 when compared with “no stress” (95% CI [1.045–1.874]) (*p* = 0.024). If one answered that they thought they were in good health, the OR of AS diagnosis was significantly low (OR 0.435, 95% CI [0.567–0.989], *p* = 0.000), and AR prevalence was also low (OR 0.749, 95% CI [0.567–0.989], *p* = 0.041) (Table 6).

### 3.6. Multiple Logistic Regression Analysis of the Association Between Allergic Diseases and Environmental Factors, Health Behaviors, and Psychosocial Risks in Korean Adolescents

To identify the environmental factors, health behaviors, and psychosocial factors affecting the occurrence of allergic diseases in Korean adolescents, a multiple logistic regression was performed with independent variables such as economic level, residential type, number of family members, smoking, obesity, and self-rated health, which were significant variables in the univariate analysis. The results are shown in Table 7.

In the AS of Korean adolescents, the OR was low when the subjective assessment of their health (self-rated health) was positive (bad vs. good; 1 vs. 0.447 (95% CI [0.28–0.712], *p* = 0.000). For AD, the OR of male students was 0.706 times (95%CI [0.501–0.994]) lower than that of female students, and a statistically significant difference (*p* = 0.046) was found. AR had a high OR when the gender was male (female vs. male; 1 vs. 1.391) (95% CI [1.007–1.922], *p* = 0.045), socioeconomic conditions were higher (lower vs. higher; 1 vs. 1.784 (95% CI [1.006–3.163)), *p* = 0.026), and stress perception was present (no vs. yes; 1 vs. 1.479 (95% CI [1.083–2.021]), *p* = 0.013).

## 4. Discussion

Few studies have explored the risk factors of the allergic diseases associated with adolescents’ health behaviors and psychosocial factors. Korean teenagers tend not to be able to visit hospitals consistently even if they have a disease, largely because of school obligations and busy schedules. As the results showed, more than a third of South Korean teenagers have one or more allergic disease.

Although the results from the multiple logistic regression showed no statistically significant difference between smoking and AS among adolescents with allergic diseases, those diagnosed with AS had the highest smoking rate. The smoking rate of adolescents diagnosed with AS was 20.6%, and that of adolescents not diagnosed with AS was 12.5%, which is statistically significant (*p* = 0.034). Compared with non-smokers, the OR of AS prevalence was 1.753 times higher for smokers. In a prospective, randomized, double-blind, crossover, placebo-controlled study examining the effects of inhaled corticosteroids in patients with mild AS, non-smoking patients experienced significantly greater improvements in morning peak expiratory flow than those who smoked (*p* = 0.0006) [7]. Smokers showed no improvement in lung function after treatment. Resistance to short-term, high-dose oral corticosteroid treatment has also been reported in patients with asthma who smoke [8]. There have been reports that if a father smokes during pregnancy, the risk of the birth child developing AS is 2.9 times higher; if a mother smokes, the risk is 1.7 times higher, and if both parents smoke, it is 3.7 times higher [9]. Therefore, education on the dangers of smoking is important for adolescents with allergic diseases. Education on smoking cessation is the most practical way to increase the effectiveness of AS treatment [10].

In this study, insufficient sleep duration was identified as a risk factor for AR. This finding aligns with previous research indicating that adolescents sleeping less than 7 h per night have an increased likelihood of developing AR [11]. Additionally, studies have shown that sleep-disordered breathing and poor sleep quality are associated with a higher risk of allergic diseases [12]. The mechanisms by which sleep deprivation increases the risk of AR are not fully understood. Some studies suggest that insufficient sleep can alter levels of inflammatory cytokines such as interleukin (IL)-1β, IL-4, IL-6, and IL-10, which may promote allergic reactions and elevate the risk of developing AR [13]. These results underscore the importance of adequate sleep for adolescents in potentially reducing the risk of AR. However, the reverse may also be true. Patients with AR are at a higher risk of developing insomnia and other sleep-related disorders. A recent meta-analysis for the adjusted OR showed that AR was associated with higher risks of nocturnal dysfunction, including insomnia, nocturnal enuresis, restless sleep, sleep-disordered breathing, obstructive sleep apnea, and snoring. In addition, there was a high prevalence of daytime dysfunction, including difficulty waking up, daytime sleepiness, morning headache, and the use of sleep medications [14]. AR causes mucous membrane edema and mucus secretion of the airway, which increases resistance in the nasal cavity and causes mouth breathing and reduced ventilation per minute, which affects carbon dioxide emissions. This mechanism is cited as one cause of sleep disorders in AR [15].

In the current study, after performing a multiple logistic regression with the independent variables for significant factors from a chi-square test analysis, the risk factors of AS were found to be cases of low self-rated health; the risk factors of AD were gender (girls) and two or less family members; and the risk factors for AR were gender (boys), higher than “lower-middle” economic levels, and stress perception. In the present study, the OR of those with more than five family members was 0.308 times lower than that of those with less than two family members, indicating that more than five family members means a lower prevalence of AD, a result which is similar to previous studies [16]. This is a result supporting the hygiene hypothesis that fewer families and cleaner environments lead to more allergic diseases [17]. In the present study, AR was significantly related to the male sex and AD to female sex. A recent systematic review also reported that one statistically significant factor in children with AR and AS is male gender [18]. According to a meta-analysis, AD persistence is significantly higher in women than in men (HR 1.15, 95% CI 1.04–1.27, *p* = 0.006) [19].

“Self-rated health” is widely used as a reliable indicator of the health of population groups, even though it is not a value that can be indicated by clinical testing. It is closely related to clinical health conditions in a way that is meaningful for individuals and allows them to evaluate their own health [20]. We found that poor self-rated health is a risk factor for AS in adolescents. Although there is limited direct evidence linking low self-rated health to the development of allergic diseases, several studies have suggested that self-rated health can serve as a predictor of future health outcomes, including the onset and progression of chronic diseases. For instance, research has shown that individuals with poorer self-rated health are more likely to develop an increasing number of chronic conditions over time [21]. Self-assessed health has been found to correlate significantly with conditions such as arthritis, high cholesterol, and lung diseases [22]. Moreover, poor self-rated health has been associated with a higher risk of developing severe chronic illnesses. It has also been identified as a significant independent predictor of both global morbidity and cause-specific morbidity, excluding cancer [23]. Conversely, there are several studies reporting that patients with allergic diseases tend to have poorer self-rated health. A study conducted on U.S. adolescents reported that those with AS symptoms had lower self-rated health compared to their peers without AS. Specifically, 13.58% of adolescents with AS and symptoms of dry cough or wheezing reported fair or poor health, compared to 7.54% of those who never had AS. Additionally, these adolescents experienced significantly impaired mental health [24].

Research indicates that self-rated health during adolescence is influenced by parental background characteristics, health status, and childhood health issues, with these associations strengthening as individuals age [25]. Adopting healthy behaviors—such as engaging in regular physical activity, obtaining sufficient sleep, maintaining a balanced diet, and avoiding alcohol and tobacco use—can enhance adolescents’ self-rated health and overall health-related quality of life [26]. Given that adolescents’ perceptions of their health can serve as predictors for the development of various diseases in early adulthood, it is crucial to implement targeted preventive measures to avert future health problems [27].

Adolescents are the most sensitive to physical changes and various stressors because of the nature of the transitional development that occurs when moving from childhood to adulthood. In the current study, stress perception was associated with an increased risk of AR. A study found that patients with higher perceived stress levels had a significantly lower quality of life related to AR compared to those with lower stress levels [28]. The exact mechanisms remain under investigation, but current evidence indicates that high stress perception can contribute to the development or worsening of AR through various immunological and behavioral pathways. Several reports have discussed the mechanisms by which stress can induce or exacerbate allergic diseases. Stress is associated with increased production of inflammatory cytokines such as IL-4, IL-5, and TNF-α [29]. Additionally, stress activates the hypothalamic–pituitary–adrenal axis, leading to elevated cortisol secretion, and stimulates the autonomic nervous system, resulting in increased levels of epinephrine and norepinephrine [30,31]. Consequently, the gene expression patterns of immune cells, such as T cells and macrophages, are altered, promoting the activation of cytokines, immune signaling pathways, and differentiation processes that facilitate allergic reactions [32]. Conversely, chronic medical condition like allergic diseases can lead to chronic stress. Allergic diseases require long-term treatment and management, which can lead to pain, fatigue, and difficulty forming relationships with friends. Also, the depression and stress caused by this may lead the disease to be poorly controlled. It has also been reported that adolescents with chronic diseases lack plans for the future and have negative thoughts about their health and family [33].

This study, being a big data analysis, offers representative insights. However, as a cross-sectional study, it has limitations in establishing causal relationships between factors and allergic diseases. To more precisely evaluate the impact of environmental factors, health behaviors, and psychosocial factors on allergic diseases, cohort studies are necessary, along with additional research into the underlying mechanisms.

## 5. Conclusions

Low self-rated health and high stress perception have been identified as risk factors for allergic diseases during adolescence. A medical environment where intervention with psychological support can be integrated is therefore needed. The importance of health risk screening and integrated preventive counseling for adolescents should be emphasized. These results can serve as the foundation for strategies through which to promote the health of teenagers with allergic diseases that require continuous treatment and care.

## Figures and Tables

**Table 1:** Relationship between environmental factors and allergic diseases in Korean adolescents.

Variable	Category	Asthma			Atopic Dermatitis			Allergic Rhinitis		
		No (*n* = 1471)	Yes (*n* = 159)	*p*-Value	No (*n* = 1447)	Yes (*n* = 183)	*p*-Value	No (*n* = 1256)	Yes (*n* = 374)	*p*-Value
		Weighted % (SE)			Weighted % (SE)			Weighted % (SE)		
Sex	Male	53 (1.5)	59.5 (4.6)	0.177	54.3 (1.5)	46.8 (4.3)	0.080	51.7 (1.7)	60.6 (3.0)	0.011 *
	Female	47 (1.5)	40.5 (4.6)		45.7 (1.5)	53.2 (4.3)		48.3 (1.7)	39.4 (3.0)	
Age	13–15	48.7 (1.6)	44.9 (4.7)	0.464	48.5 (1.5)	47.1 (4.3)	0.760	48 (1.8)	49.7 (2.9)	0.640
	16–18	51.3 (1.6)	55.1 (4.7)		51.5 (1.5)	52.9 (4.3)		52 (1.8)	50.3 (2.9)	
Residence	Urban	81.7 (2.7)	84.5 (3.8)	0.483	81.6 (2.6)	86.7 (3.9)	0.199	81.2 (2.9)	84.8 (3.0)	0.318
	Rural	18.3 (2.7)	15.5 (3.8)		18.4 (2.6)	13.3 (3.9)		18.8 (2.9)	15.2 (3.0)	
Family composition	2 generations	83.4 (1.5)	81.5 (3.8)	0.632	82.8 (1.4)	87.1 (3.5)	0.270	82.5 (1.6)	85.9 (2.3)	0.211
	3 generations	16.6 (1.5)	18.5 (3.8)		17.2 (1.4)	12.9 (3.5)		17.5 (1.6)	14.1 (2.3)	
Socioeconomic status	Lower	14.6 (1.5)	25.6 (4.8)	0.004 *	15.5 (1.6)	18.5 (3.7)	0.650	16.4 (1.7)	13.0 (2.5)	0.011 *
	Lower-middle	29.1 (1.8)	28.5 (4.8)		29.3 (1.9)	24.1 (4.4)		30.2 (2.0)	24.7 (2.9)	
	Upper-middle	28.8 (1.7)	16.2 (3.4)		27.5 (1.7)	28.4 (4.0)		28.1 (1.7)	25.6 (3.1)	
	Upper	27.5 (1.7)	29.4 (4.5)		27.7 (1.8)	29.0 (3.9)		25.3 (1.9)	36.6 (3.3)	
Type of house	Apartment	35.7 (1.6)	27.3 (3.7)	0.048 *	34.3 (1.6)	40.0 (4.4)	0.219	33.4 (1.8)	40.3 (3.2)	0.064
	General housing	64.3 (1.6)	72.7 (3.7)		65.7 (1.6)	60.0 (4.4)		66.6 (1.8)	59.7 (3.2)	
Number in family	Less than 2	3.7 (0.6)	9 (2.7)	0.076	3.8 (0.6)	7.6 (2.6)	0.022 *	4.2 (0.7)	4 (1.4)	0.274
	3	20.5 (1.5)	20.6 (4.1)		20.7 (1.5)	19.1 (3.6)		20 (1.5)	22.7 (2.7)	
	4	46.8 (1.8)	42.1 (4.7)		45.2 (1.9)	53.9 (4.7)		45.5 (2.0)	49.3 (3.1)	
	More than 5	29 (1.8)	28.3 (4.5)		30.3 (1.9)	19.4 (3.4)		30.3 (1.9)	23.9 (2.6)	

*p* values (* < 0.05) are shown for chi-square tests.

**Table 2:** Relationship between health behaviors and allergic diseases in Korean adolescents.

Variable		Category	Asthma			Atopic Dermatitis			Allergic Rhinitis		
			No (*n* = 1471)	Yes (*n* = 159)	*p*-Value	No (*n* = 1447)	Yes (*n* = 183)	*p*-Value	No (*n* = 1256)	Yes (*n* = 374)	*p*-Value
			Weighted % (SE)			Weighted % (SE)			Weighted % (SE)		
Drinking		Yes	32.1 (1.7)	39.7 (5.1)	0.145	32.6 (1.7)	34.9 (4.6)	0.619	33.8 (1.9)	29.5 (2.8)	0.201
		No	67.9 (1.7)	60.3 (5.1)		67.4 (1.7)	65.1 (4.6)		66.2 (1.9)	70.5 (2.8)	
Tobacco smoking		Yes	12.5 (1.1)	20.6 (4.3)	0.034 *	13.3 (1.1)	14.2 (3.3)	0.785	13.1 (1.2)	13.9 (2.5)	0.781
		No	87.5 (1.1)	79.4 (4.3)		86.7 (1.1)	85.8 (3.3)		86.9 (1.2)	86.1 (2.5)	
Influenza vaccination		Yes	75.4 (1.5)	69.4 (4.5)	0.170	74.3 (1.1)	79.2 (3.5)	0.207	74.2 (1.6)	77.1 (2.6)	0.359
		No	24.6 (1.5)	30.6 (4.5)		25.7 (1.1)	20.8 (3.5)		25.8 (1.6)	22.9 (2.6)	
Obesity (BMI ≥ 25 kg/m^2^)		Yes	13.6 (1.1)	20.4 (4.0)	0.056	14.5 (1.1)	13.0 (2.9)	0.635	13.3 (1.2)	17.9 (2.4)	0.057
		No	86.4 (1.1)	79.6 (4.0)		85.5 (1.1)	87.0 (2.9)		86.7 (1.2)	82.1 (2.4)	
Physical Activity	Intense Exercise	Yes	28.1 (1.4)	33.3 (4.8)	0.300	29.2 (1.4)	23.9 (3.3)	0.172	27.9 (1.5)	31.4 (2.7)	0.267
		No	71.9 (1.4)	66.7 (4.8)		71.8 (1.4)	76.1 (3.3)		72.1 (1.5)	68.6 (2.7)	
	Moderate Exercise	Yes	7.4 (0.8)	8.2 (2.7)	0.764	7.4 (0.8)	7.6 (2.2)	0.922	6.9 (0.8)	9.5 (1.8)	0.146
		No	92.6 (0.8)	91.8 (2.7)		92.6 (0.8)	92.4 (2.2)		93.1 (0.8)	90.5 (1.8)	
	Walking Exercise	Yes	53.2 (1.6)	48.9 (4.9)	0.409	52.5 (1.7)	54.8 (4.3)	0.630	51.6 (1.6)	57 (3.2)	0.118
		No	46.8 (1.6)	51.1 (4.9)		47.5 (1.7)	45.2 (4.3)		48.4 (1.6)	43 (3.2)	
	Strengthening Exercise	≥4 days	91.1 (1.0)	91.8 (2.7)	0.796	90.8 (1)	93.6 (2.2)	0.294	91.4 (1)	90.2 (1.9)	0.538
		<4 days	8.9 (1.0)	8.2 (2.7)		9.2 (1)	6.4 (2.2)		8.6 (1)	9.8 (1.9)	
	Flexibility Exercise	≥4 days	86.9 (1.1)	87.4 (3.3)	0.879	86.9 (1.1)	86.1 (3.3)	0.810	87.3 (1.1)	85.4 (2.2)	0.387
		<4 days	13.1 (1.1)	12.6 (3.3)		13.1 (1.1)	13.9 (3.3)		12.7 (1.1)	14.6 (2.2)	
Hours of sleep		≥7 h	35.5 (1.7)	38.9 (4.7)	0.500	35.8 (1.7)	36.0 (4.0)	0.979	37.9 (1.8)	28 (2.8)	0.002 *
		<7 h	64.5 (1.7)	61.1 (4.7)		64.2 (1.7)	64.0 (4.0)		62.1 (1.8)	72 (2.8)	
25(OH) Vitamin D		<20 ng/mL	83 (2)	82.2 (5.6)	0.897	82.2 (2.1)	88.5 (4.2)	0.243	84.5 (2.1)	76.5 (4.6)	0.081
		≥20 ng/mL	17 (2)	17.8 (5.6)		17.8 (2.1)	11.5 (4.2)		15.5 (2.1)	28.5 (4.6)	

*p* values (* < 0.05) are shown for chi-square tests.

**Table 3:** Relationship between psychosocial factors and allergic diseases in Korean adolescents.

Variable	Category	Asthma			Atopic Dermatitis			Allergic Rhinitis		
		No (*n* = 1471)	Yes (*n* = 159)	*p*-Value	No (*n* = 1447)	Yes (*n* = 183)	*p*-Value	No (*n* = 1256)	Yes (*n* = 374)	*p*-Value
		Weighted % (SE)			Weighted % (SE)			Weighted % (SE)		
Absence from school last month	Yes	4.5 (0.7)	6.2 (2.6)	0.443	4.8 (0.7)	3.3 (1.9)	0.494	4.6 (0.8)	5 (1.4)	0.752
	No	95.5 (0.7)	93.8 (2.6)		95.2 (0.7)	96.7 (1.9)		95.4 (0.8)	95 (1.4)	
Stress perception	Yes	26.1 (1.5)	28.6 (4.1)	0.543	25.9 (1.5)	29.8 (4.1)	0.361	25.1 (1.5)	30.8 (2.7)	0.047 *
	No	73.9 (1.5)	71.4 (4.1)		74.1 (1.5)	70.2 (4.1)		74.9 (1.5)	69.2 (2.7)	
Suicidal ideation	Yes	14.1 (1.1)	18.6 (3.7)	0.216	14.5 (1.1)	14.8 (3.1)	0.916	13.8 (1.2)	17 (2.4)	0.217
	No	85.9 (1.1)	81.4 (3.7)		85.5 (1.1)	85.2 (3.1)		86.2 (1.2)	83 (2.4)	
Depression symptom experience	Yes	11 (1.1)	10.8 (3.1)	0.952	11.4 (1.0)	7.5 (2.4)	0.166	10.5 (1.2)	12.8 (2.3)	0.372
	No	89 (1.1)	89.2 (3.1)		88.6 (1.0)	92.5 (2.4)		89.5 (1.2)	87.2 (2.3)	
Mental health counseling	Yes	3.9 (0.6)	4.9 (1.9)	0.588	3.8 (0.6)	5.8 (2.0)	0.273	3.7 (0.6)	5 (1.3)	0.377
	No	96.1 (0.6)	95.1 (1.9)		96.2 (0.6)	94.2 (2.0)		96.3 (0.6)	95 (1.3)	
Self-rated health	Healthy	61.3 (1.6)	41.1 (5.0)	0.000 *	59.7 (1.7)	56.3 (4.3)	0.454	60.7 (1.7)	54.3 (3.2)	0.062
	Unhealthy	38.7 (1.6)	58.9 (5.0)		40.3 (1.7)	43.7 (4.3)		39.3 (1.7)	45.7 (3.2)	

*p* values (* < 0.05) are shown for chi-square tests.

**Table 4:** Odd ratios of allergic diseases in Korean adolescents for environmental factors using binary logistic regression analysis.

Variable	Category	Asthma		Atopic Dermatitis		Allergic Rhinitis	
		Age, Sex-Adjusted OR (95% CI)	*p*-Value	Age, Sex-Adjusted OR (95% CI)	*p*-Value	Age, Sex-Adjusted OR (95% CI)	*p*-Value
Sex	Female	1	0.185	1	0.083	1	0.036 *
	Male	1.3 (0.881, 1.919)		0.741 (0.528, 1.041)		1.441 (1.082, 1.916)	
Age	13–15	1	0.453	1	0.187	1	0.916
	16–18	1.169 (0.778, 1.757)		1.566 (0.804, 3.050)		1.034 (0.558, 1.915)	
Residence	Urban	1	0.529	1	0.168	1	0.367
	Rural	1.197 (0.683, 2.097)		1.508 (0.841, 2.702)		1.258 (0.764, 2.071)	
Family composition	2 generations	1	0.589	1	0.246	1	0.220
	3 generations	1.16 (0.680, 1.980)		0.70 (0.390, 1.280)		0.78 (0.520, 1.160)	
Socioeconomic status	Lower	1	0.016 *	1	0.922	1	0.012 *
	Lower-middle	0.59 (0.316, 1.110)		0.84 (0.417, 1.693)		1.125 (0.659, 1.920)	
	Upper-middle	0.333 (0.170, 0.652)		1.002 (0.522, 1.925)		1.195 (0.665, 2.146)	
	Upper	0.562 (0.313, 1.009)		1.008 (0.532, 1.910)		1.965 (1.129, 3.419)	
Type of house	General housing	1	0.048 *	1	0.218	1	0.063
	Apartment	0.679 (0.460, 1.000)		1.276 (0.866, 1.881)		1.344 (0.983, 1.836)	
Numbers in family	Less than 2	1	0.077	1	0.006 *	1	0.334
	3	0.394 (0.163, 0.952)		0.478 (0.195, 1.168)		1.164 (0.512, 2.648)	
	4	0.367 (0.173, 0.778)		0.595 (0.260, 1.359)		1.125 (0.505, 2.505)	
	More than 5	0.408 (0.180, 0.925)		0.308 (0.139, 0.681)		0.846 (0.371, 1.929)	

*p* values (* < 0.05) are shown for binary logistic regression of differences between categories about the variable.

**Table 5:** Odd ratios of allergic diseases in Korean adolescents for health behavior using binary logistic regression analysis.

Variable	Category	Asthma		Atopic Dermatitis		Allergic Rhinitis	
		Age, Sex-Adjusted OR (95% CI)	*p*-Value	Age, Sex-Adjusted OR (95% CI)	*p*-Value	Age, Sex-Adjusted OR (95% CI)	*p*-Value
Alcohol drinking	No	1	0.227	1	0.443	1	0.196
	Yes	1.384 (0.817, 2.345)		1.189 (0.764, 1.851)		0.796 (0.563, 1.126)	
Tobacco smoking	No	1	0.006 *	1	0.563	1	0.922
	Yes	1.753 (0.974, 3.155)		1.184 (0.668, 2.096)		1.025 (0.628, 1.673)	
Influenza vaccination	No	1	0.153	1	0.221	1	0.223
	Yes	1.374 (0.888, 2.125)		0.766 (0.499, 1.174)		0.816 (0.588, 1.132)	
Obesity	No	1	0.072	1	0.705	1	0.070
	Yes	1.595 (0.959, 2.653)		0.905 (0.54, 1.517)		1.399 (0.973, 2.011)	
Physical activity							
Intense exercise	No	1	0.423	1	0.307	1	0.655
	Yes	1.218 (0.751, 1.976)		0.811 (0.543, 1.212)		1.069 (0.797, 1.433)	
Moderate exercise	No	1	0.826	1	0.822	1	0.221
	Yes	1.088 (0.512, 2.312)		1.078 (0.558, 2.084)		1.349 (0.836, 2.177)	
Walking exercise	No	1	0.386	1	0.589	1	0.137
	Yes	0.835 (0.556, 1.255)		1.109 (0.761, 1.616)		1.228 (0.936, 1.611)	
Weight training	<4 days	1	0.591	1	0.455	1	0.898
	≥4 days	1.247 (0.557, 2.792)		1.339 (0.623, 2.878)		0.970 (0.603, 1.560)	
Flexibility exercise	<4 days	1	0.889	1	0.843	1	0.309
	≥4 days	1.045 (0.558, 1.958)		0.944 (0.534, 1.671)		0.826 (0.572, 1.194)	
Sleeping hours	<7 h	1	0.473	1	0.959	1	0.000 *
	≥7 h	1.178 (0.754, 1.840)		1.01 (0.688, 1.483)		0.601 (0.441, 0.820)	

*p* values (* < 0.05) are shown for binary logistic regression.

**Table 6:** Odd ratios of allergic diseases in Korean adolescents for psychosocial factors using binary logistic regression analysis.

Variable	Category	Asthma		Atopic Dermatitis		Allergic Rhinitis	
		Age, Sex-Adjusted OR (95% CI)	*p*-Value	Age, Sex-Adjusted OR (95% CI)	*p*-Value	Age, Sex-Adjusted OR (95% CI)	*p*-Value
Limited activity	No	1	0.362	1	0.425	1	0.540
	Yes	1.513 (0.621, 3.684)		0.63 (0.203, 1.961)		1.221 (0.645, 2.311)	
Stress perception	No	1	0.486	1	0.436	1	0.024 *
	Yes	1.161 (0.762, 1.769)		1.184 (0.775, 1.809)		1.399 (1.045, 1.874)	
Suicidal ideation	No	1	0.147	1	0.889	1	0.090
	Yes	1.495 (0.868, 2.576)		0.963 (0.563, 1.647)		1.406 (0.948, 2.086)	
Depression symptom experience	No	1	0.988	1	0.137	1	0.258
	Yes	0.995 (0.517, 1.917)		0.608 (0.315, 1.172)		1.318 (0.816, 2.128)	
Mental health counseling	Yes	1	0.496	1	0.357	1	0.249
	No	0.733 (0.300, 1.791)		0.685 (0.306, 1.533)		0.668 (0.336, 1.327)	
Self-rated health	Bad	1	0.000 *	1	0.463	1	0.041 *
	Good	0.435 (0.281, 0.675)		0.871 (0.601, 1.261)		0.749 (0.567, 0.989)	

*p* values (* < 0.05) are shown for binary logistic regression.

**Table 7:** Multiple logistic regression analysis for the association between allergic diseases and environmental factors, health behaviors, and psychosocial risks in Korean adolescents.

Variable	Category	Asthma		Atopic Dermatitis		Allergic Rhinitis	
		OR (95% CI)	*p*-Value	OR (95% CI)	*p*-Value	OR (95% CI)	*p*-Value
Gender	Female			1	0.046 *	1	0.045 *
	Male			0.706 (0.501, 0.994)		1.391 (1.007, 1.922)	
Number of people in family	Less than 2			1	0.006 *		
	3			0.296 (0.133, 0.661)			
	4			0.555 (0.241, 1.278)			
	More than 5			0.445 (0.182, 1.084)			
Socio -economic status	Lower					1	0.026 *
	Lower-middle					1.05 (0.603, 1.796)	
	Upper-middle					1.073 (0.583, 1.976)	
	Upper					1.784 (1.006, 3.163)	
Stress perception	No					1	0.013 *
	Yes					1.479 (1.083, 2.021)	
Self-rated health	Bad	1	0.000 *				
	Good	0.447 (0.280, 0.712)					

*p* values (* < 0.05) are shown for multiple logistic regression. Factors are socioeconomic status, type of house, number of people in family, smoking, obesity, and self-rated health input.

## Data Availability

Restriction apply to the availability of these data. The data were obtained from the KNHNES conducted by the Korea Disease Control and Prevention Agency (KDCA) and are available at https://knhanes.kdca.go.kr/knhanes/main.do (accessed on 16 March 2025) with the permission of the KDCA.

## Abbreviations

The following abbreviations are used in this manuscript:

KNHNES	Korean National Health and Nutrition Examination Survey
AD	Atopic dermatitis
AR	Allergic rhinitis
AS	Asthma
